# Carcinogenesis and management of human papillomavirus-associated cervical cancer

**DOI:** 10.1007/s10147-023-02337-7

**Published:** 2023-06-09

**Authors:** Misako Kusakabe, Ayumi Taguchi, Kenbun Sone, Mayuyo Mori, Yutaka Osuga

**Affiliations:** 1 Department of Obstetrics and Gynecology, Graduate School of Medicine, The University of Tokyo, 7-3-1 Hongo Bunkyo-ku, Tokyo, 113-8655 Japan; 2 Laboratory of Human Single Cell Immunology, World Premier International Immunology Frontier Research Center (WPI-IFReC), Osaka University, 3-1 Yamadaoka, Suita, Osaka, 565-0871 Japan

**Keywords:** Human papillomavirus, Cervical cancer, Squamocolumnar junction

## Abstract

Approximately 95% of cervical cancer are caused by human papillomavirus (HPV) infection. Although it is estimated that HPV-associated cervical cancer will decrease with the widespread use of HPV vaccine, it may take time for HPV-associated cervical cancer to be eliminated. For the appropriate management of HPV-associated cervical cancer, it is important to understand the detailed mechanisms of cervical cancer development. First, the cellular origin of most cervical cancers is thought to be cells in the squamocolumnar junction (SCJ) of the uterine cervix. Therefore, it is important to understand the characteristics of SCJ for cervical cancer screening and treatment. Second, cervical cancer is caused by high risk HPV (HR-HPV) infection, however, the manner of progression to cervical cancer differs depending on the type of HR-HPV: HPV16 is characterized by a stepwise carcinogenesis, HPV18 is difficult to detect in precancerous lesions, and HPV52, 58 tends to remain in the state of cervical intraepithelial neoplasia (CIN). Third, in addition to the type of HPV, the involvement of the human immune response is also important in the progression and regression of cervical cancer. In this review, we demonstrate the carcinogenesis mechanism of HPV-associated cervical cancer, management of CIN, and the current treatment of CIN and cervical cancer.

## Epidemiology of cervical cancer and the introduction of HPV vaccination

Human papillomavirus (HPV) infection is the main cause of cervical cancer. Despite the recent introduction of an HPV vaccination, cervical cancer remains the fourth most commonly diagnosed cancer among women worldwide and the fourth leading cause of cancer-related death [1]. Approximately 10,000 patients were newly diagnosed with cervical cancer in 2019 in Japan, and the number is growing slightly [2]. Cervical cancer is classified into two major histologic types, squamous cell carcinoma (72.6%) and adenocarcinoma (21.8%) [3] and the 5-year survival rates by histologic type are 81.4% for squamous cell carcinoma and 75.8% for adenocarcinoma, with adenocarcinoma having a worse prognosis than squamous cell carcinoma [3]. More than 80% of cervical adenocarcinoma are HPV associated, but 10–15% of them, such as gastric-type adenocarcinoma and clear cell carcinoma, have been reported to be HPV independent [4]. However, squamous cell carcinomas, which comprise the majority of cervical cancers are mostly caused by HPV infection, and approximately 95% of cervical cancers are HPV-positive [5]. There are other rare histological types of HPV-associated cervical cancer, such as small cell carcinoma and glassy cell carcinoma [6, 7].

It is expected that HPV-associated cervical cancer will decrease with the widespread use of HPV prophylactic vaccines. In Sweden, where the HPV prophylactic vaccine was introduced at an early age, administration of the vaccine had a significant effect on cervical cancer prevention, with incidence ratios of 0.12 (95% CI 0.00–0.34) and 0.47 (95% CI 0.27–0.75) for women who received the HPV vaccine before age of 17 and between ages of 17 and 30, respectively [8]. In Australia, if current levels of vaccination and screening coverage are maintained the annual incidence of cervical cancer is estimated to fall below four cases per 100,000 women by 2028 (range 2021–35) [9].

In Japan, a publicly-funded HPV vaccination program for girls aged 13–16 began in 2010, and HPV vaccination was included in the national immunization program for 12–16 aged girls in April 2013. However, soon after the introduction of HPV vaccination, the Ministry of Health, Labor and Welfare (MHLW) in Japan decided to suspend proactive recommendation of the vaccine as there were repeated reports of diverse symptoms, including motor impairment and chronic pain, from girls who had been vaccinated. As a result, the prevalence of HPV prophylactic vaccines has fallen below 1% [10]. In January 2014, after investigating these adverse events, MHLW concluded that there was no evidence to suggest a causal relationship between the HPV vaccine and adverse events, however, MHLW did not resume its proactive recommendation. Since then, the Nagoya Study has also reported no association between the HPV vaccine and reported adverse events [11]. Considering these results, the Japanese government decided to resume the proactive recommendation of the HPV vaccine in April 2022.

To prevent cervical cancer, in addition to primary prevention by HPV vaccine, secondary prevention with early detection through screening is also important. However, the low examination rate for cervical cancer screening in Japan has also been an issue. The ideal screening rate for cervical cancer elimination is 70% [12], however, in Japan it is only around 40% [13]. As a screening method for cervical cancer, a combination of cytological diagnosis by Pap smear and HPV testing is becoming popular worldwide. In general, cytology alone has low sensitivity and a high false-negative rate [14] but combining it with HPV testing increases sensitivity [15] and reduces costs by extending the interval between examinations and completing the examinations earlier. Although the 2012 guidelines of the American Cancer Society, the American Society for Colposcopy and Cervical Pathology, and American Society for Clinical Pathology recommended a combination of cytology and HPV testing (co-testing) every 5 years between the ages of 30 and 65 [16], the latest American Cancer Society guidelines for 2020 recommend FDA-approved HPV testing every 5 years after age 25 [17]. Consequently, HPV testing is likely to become the main screening method for cervical cancer in the future. While the introduction of the HPV vaccine in the U.S. might have led to a decrease in false-positive results for precancerous lesions in HPV testing, the usefulness of cytological-based screening is still supported in Japan where the introduction of the vaccine has been delayed. According to the Japanese guideline for cervical cancer screening at National Cancer Center [18], both cytology-based screening and HPV testing alone are recommended.

## The cellular origin of cervical cancer and HPV-associated carcinogenesis

**Fig. 1:**
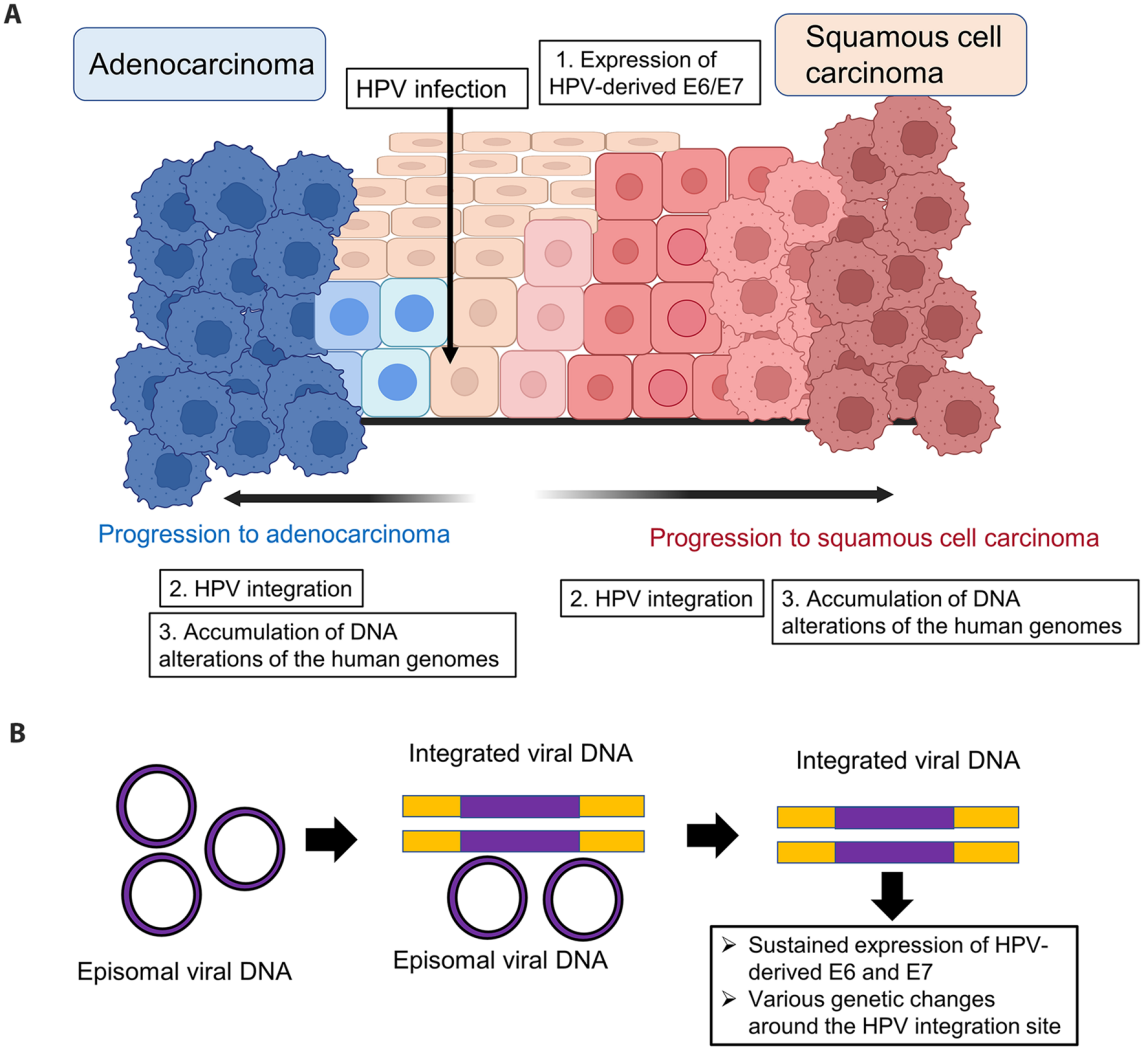
Carcinogenesis mechanism of cervical cancer. **A** Carcinogenesis mechanism of uterine squamous cell carcinoma and adenocarcinoma. Human papillomavirus (HPV) infects cells in the basal layer of the cervix and causes carcinogenesis over the years. Squamous cell carcinoma develops through a stepwise progression of its precancer lesions (cervical intraepithelial neoplasia, CIN). The steps to carcinogenesis include expression of HPV-derived oncogenes, E6/E7, HPV integration, and accumulation of human oncogene mutations. The figure was created with BioRender.com. **B** Changes in HPV genomic status. During persistent HPV infection, the integration of the HPV genome into the human genome occurs. HPV integration contributes to the sustained expression of HPV-derived E6/E7 as well as induces various genetic changes such as amplification of oncogenes, chromosomal rearrangements, and chromosomal instability around the HPV integration site

More than 200 genotypes of HPV have been reported [19]. Of these, only the mucosal type infects the uterine cervix. The International Agency for Research on Cancer (IARC) classifies HPV as Groups 1–4 based on the risk of carcinogenesis [20] and Group 1 or Group 2A with a high risk of carcinogenesis includes 13 types of HPV: HPV types 16, 18, 31, 33, 35, 39, 45, 51, 52, 56, 58, 59, and 68. In particular, HPV types 16 and 18 are responsible for 70% of the HPV genotypes detectable in cervical cancer [21]. When HPV infects target cells, HPV-derived oncogenes E6 and E7 inactivate the tumor suppressor genes *p53* and *pRb,* respectively, leading to apoptosis resistance and abnormal cell proliferation [22]. In approximately 90% of patients with HPV infection, the immune response spontaneously eliminates HPV-infected cells, but some patients develop a persistent infection, which is discussed in more detail in Chapters 3 and 4. A fraction of patients who develop persistent infections will progress to cervical cancer through the following steps. During persistent HPV infection, integration of the HPV genome into the human genome occurs, resulting in sustained expression of HPV-derived E6 and E7. HPV integration not only contributes to the persistent expression of HPV-derived E6/E7, but also induces various genetic alterations such as amplification of oncogenes, chromosomal rearrangements, and chromosomal instability around the HPV integration site [23, 24] (Fig. 1A, B). After accumulation of DNA alterations in the host (human) genomes, HPV-infected cells eventually progress to cervical cancer.

**Fig. 2:**
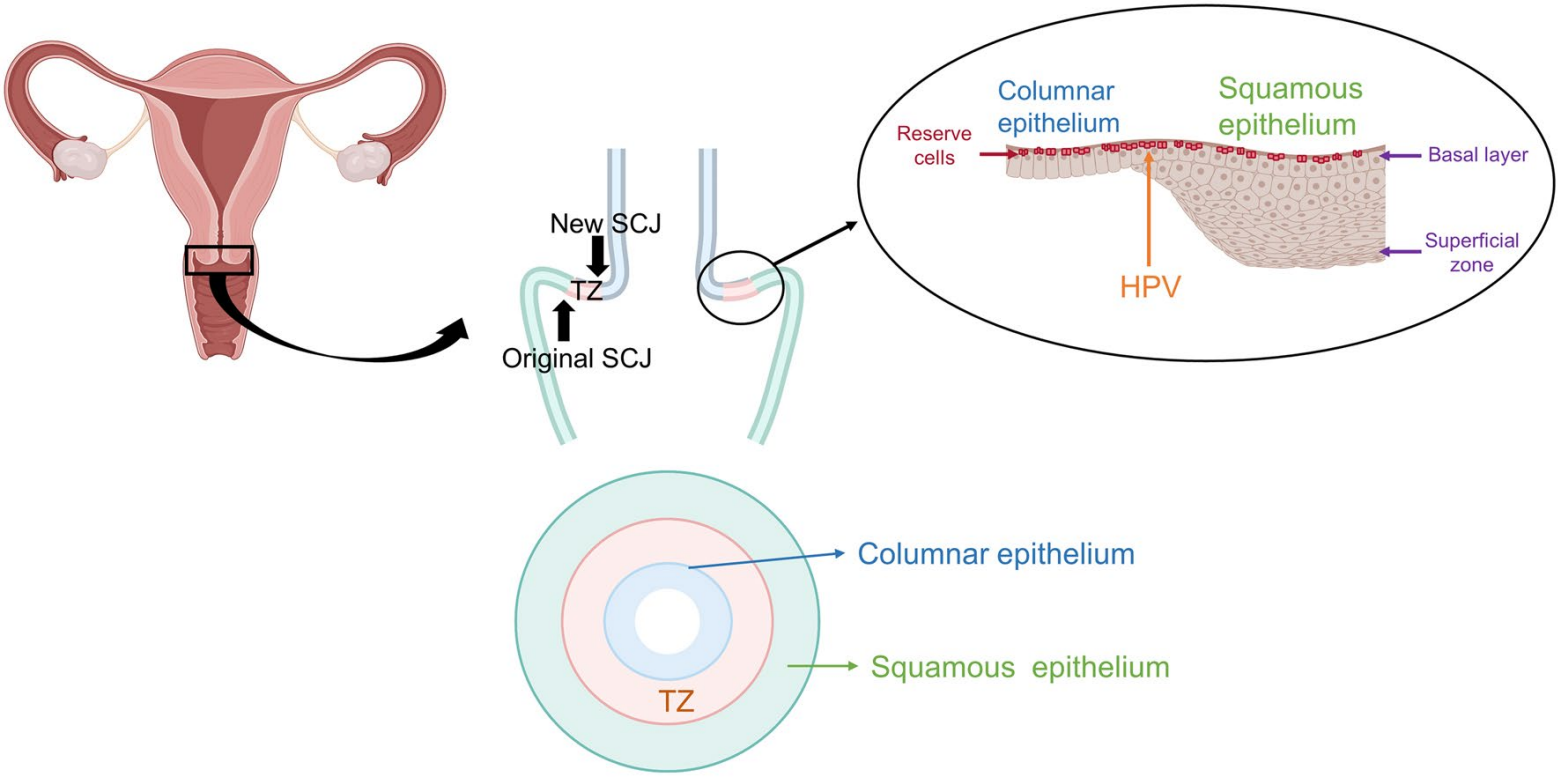
Structure of the uterine cervix. The uterine cervix consists of three distinct types of epithelia. The boundary between the squamous and columnar epithelium is referred to as the squamocolumnar junction (SCJ). Reserve cells are located in the basal layer of the cervical SCJ. *SCJ* squamocolumnar junction, *TZ* transformation zone. The figure was created with BioRender.com

The uterine cervix consists of three distinct types of epithelium: the stratified squamous epithelium of the outer cervix, the monolayered columnar epithelium of the inner cervix, and the transformation zone (TZ) between the two (Fig. 2). The boundary between the squamous and columnar epithelium is called the squamocolumnar junction (SCJ) (Fig. 2).

In women of reproductive age, vaginal acid stimulation causes squamous transformation of the columnar epithelium of the uterine cervix. Stem cells (reserve cells) in the SCJ are thought to be involved in this transformation [25, 26]. The area between the original SCJ and the new SCJ is known as the transformation zone (TZ) [27]. There are various theories regarding the cellular origin of cervical cancer, including the theory that HPV infects SCJ cells as well as the theory that HPV infects reserve cells [26]. These two theories are not exclusive. The SCJ is a cuboidal cell that borders the squamous and columnar epithelium, and cells in this area are thought to have the ability to differentiate into squamous or columnar epithelium. SCJ cells are characterized by the expression of SCJ markers such as cytokeratin 7 (CK7), anterior gradient (AGR) 2, cluster differentiation (CD) 63, matrix metalloproteinase (MMP) 7 and guanine deaminase (GDA) [28]. SCJ was considered as the cellular origin of cervical cancer for the following two reasons. First, these SCJ markers are expressed in cervical cancer and its precursor lesion, cervical intraepithelial neoplasia (CIN) [28], and second, cervical cancer and CIN frequently originate from the SCJ region. Therefore, close monitoring of the SCJ region is extremely important in the examination of cervical lesions. In particular, the accuracy of colposcopy decreases with age because TZ often migrates into the cervical canal [29]. Therefore, understanding the pathogenesis of HPV-derived cervical cancer and the characteristics of TZ is essential during primary screening and close examination of HPV-infected cervical lesions.

## Natural history of HPV-related cervical lesions according to HPV genotypes

HPV-associated cervical cancer includes squamous cell carcinoma, adenocarcinoma (including adenosquamous carcinoma), and small cell carcinoma. Squamous cell carcinoma, which accounts for approximately 70% of all cervical cancers, develops through CINs. CIN can be classified into three stages, CIN1, CIN2, and CIN3, or two stages as low-grade squamous intraepithelial lesions (LSIL, CIN1) and high-grade squamous intraepithelial lesions (HSIL, CIN2-3) [30]. HSIL is characterized by positive p16 due to HPV-induced cell cycle activation. In NCCN guideline 2022, HSIL is considered a precancerous lesion and HSIL is indicated for treatments such as cervical conization and laser vaporization [31]. The precancerous lesions of adenocarcinoma are adenocarcinoma in situ (AIS), which, unlike CIN, does not take the form of gradual progression. Unlike squamous cell carcinoma and adenocarcinoma with precancerous lesions, the precancerous lesions have not been identified in small cell carcinoma and the cell of origin is still unknown.

**Fig. 3:**
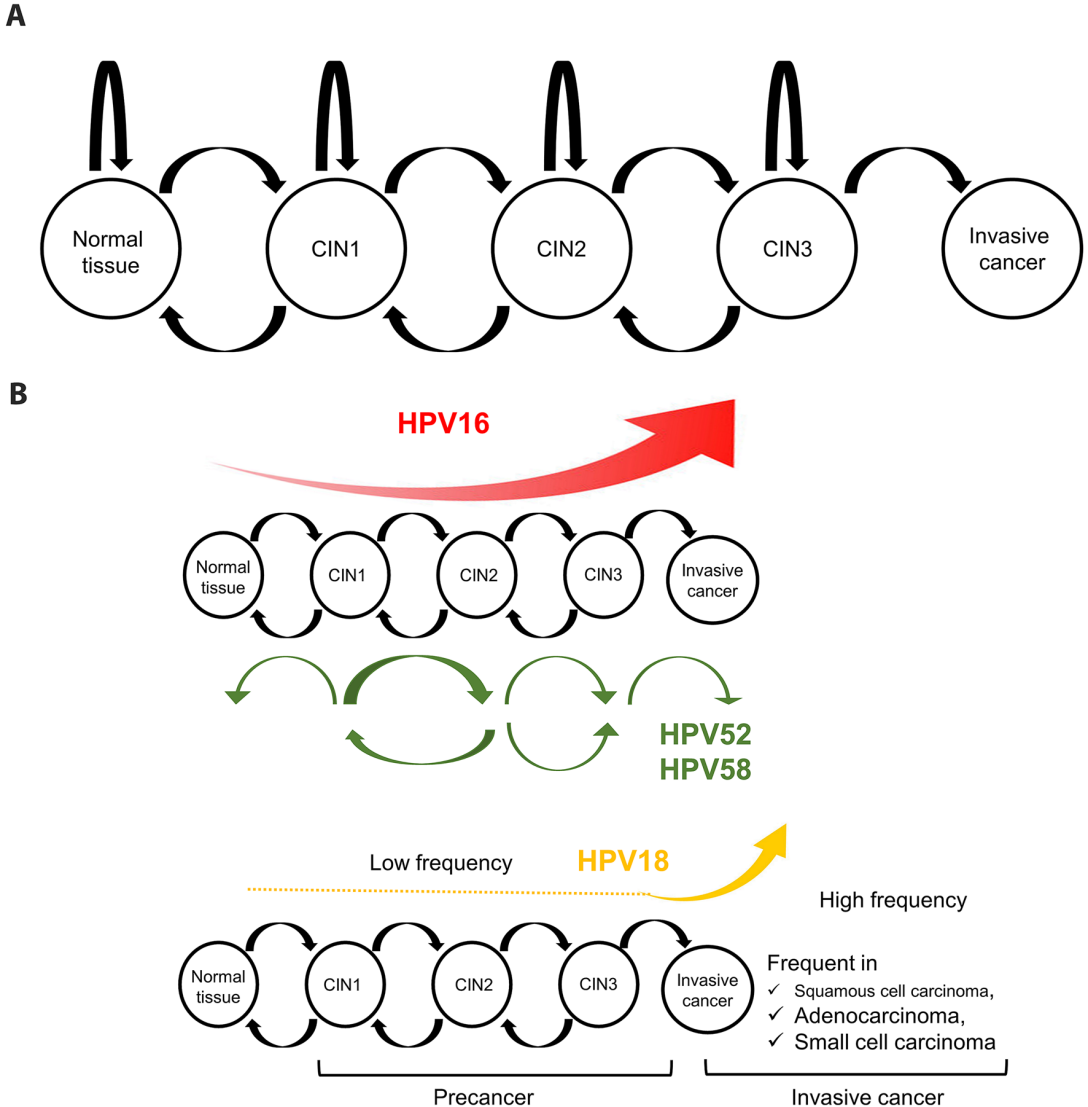
The natural history of HPV-associated cervical lesions. **A** Bidirectional transition of cervical intraepithelial neoplasia (CIN). The natural history of CIN shows a bidirectional transition between different states (normal, CIN1, CIN2, and CIN3). **B** HPV-genotype-specific characteristics of cervical cancer development. HPV16-related lesion is characterized by stepwise progression to CIN3 or higher, whereas HPV52 and HPV58-related lesions are characterized by their persistency between CIN1 and CIN2. HPV18 is frequently detected in rate in cervical cancer, but its detection rate in precancerous lesions is low. *HPV* human papillomavirus, *CIN* cervical intraepithelial neoplasia

In general, HPV infection is temporary and about 90% of HPV infections are spontaneously eliminated within 2 years and do not develop into persistent infections [32]. However, a small proportion of these patients develop cervical cancer through persistent HPV infection. Infected HPV genotype is known to be involved in the progression of CIN. Among the 13 high-risk HPV types, 7 HPV types, HPV types 16, 18, 31, 33, 35, 52, and 58 have an approximately 20% rate of progression to CIN3 or higher within 5 years and are considered particularly high risk [33]. The progression of CIN is generally considered to be a gradual process from HPV infection through CIN1 to CIN2/3 to cervical cancer over the years. This change is not unidirectional but is characterized by repeated progression and regression and a bidirectional transition [34] (Fig. 3A). Recently, the Markov model, a statistical analysis method that takes into account the natural history of CIN, which is characterized by bidirectional transition, has been used to estimate the prognosis of CIN. Using the Markov model, it became clear that among these seven particularly high-risk types, the form of CIN progression differs depending on the HPV genotype; HPV16 is characterized by stepwise progression to CIN3 or higher, whereas HPV52 and HPV58 are characterized by persistent CIN1-2 [35, 36] (Fig. 3B). It is also important to note that among high-risk HPVs, HPV18 has characteristics not found in other types: HPV18 has a detection rate of approximately 25% in cervical cancer among Japanese [21], which is the second most common following HPV16, but its probability of being found in precancerous lesions is lower than with other high-risk HPVs, only about 5% [37] (Fig. 3B). One of the reasons for this is related to the histological characteristics of HPV18, which is more common in adenocarcinomas and small cell carcinomas among cervical cancers, and it is more difficult to detect precancerous lesions in these histological types than in squamous cell carcinomas [38, 39]. However, the low detection rate of HPV18 in precancerous lesions cannot be explained by histological characteristics alone, and some unique mechanisms are thought to be involved in HPV18 carcinogenesis [37, 40]. A study analyzing the cellular origin of mixed carcinoma of the uterine cervix reported that maintenance of stem cell-like components is important for HPV18 carcinogenesis [40]. Although further studies are needed to elucidate HPV18-associated carcinogenesis, the low detection rate of precancerous lesions and the high incidence of invasive cancer suggest that HPV18-positive patients require more careful management than patients with other types of infection.

## Evasion of HPV-infected cells from human immunity

**Fig. 4:**
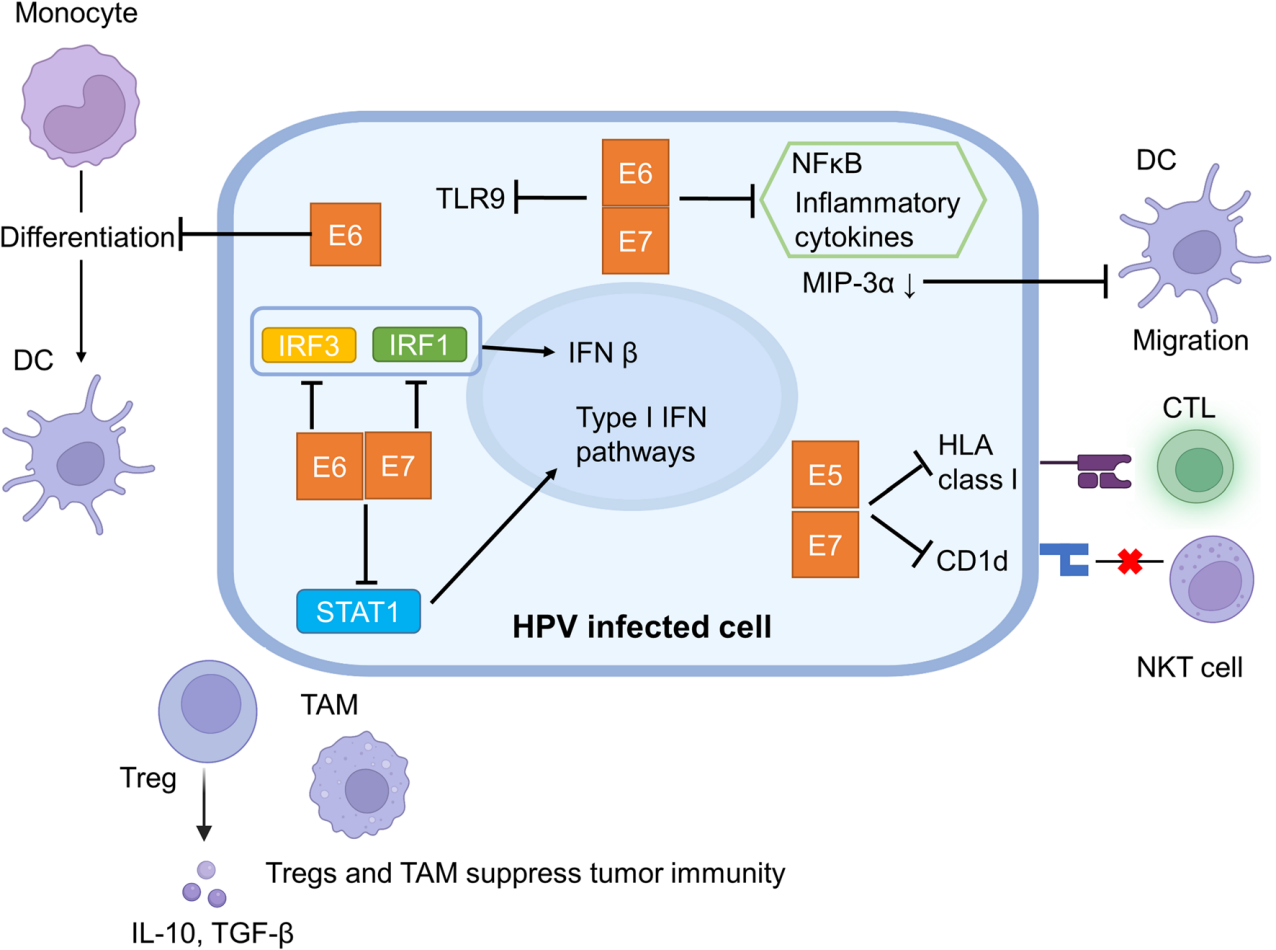
Immune escape mechanisms of HPV-infected cells. *TLR* toll-like receptors, *DC* Dendritic cell, *TAM* tumor-associated macrophage, *CTL* cytotoxic T lymphocyte, *Treg* regulatory T cell, *IFN* interferon, *IRF* interferon regulatory factor, *NKT* natural killer T cell, *IL-10* interleukin-10, *TGF* tumor growth factor, *NFκB* nuclear factor-kappa B, *MIP* macrophage inflammatory protein, *HLA* human leukocyte antigen, *STAT1* signal transducer and activator of transcription 1. The figure was created with BioRender.com

Evasion of human immune surveillance mechanisms is essential for persistent HPV infection to occur and its progression to cervical cancer. HPV infection of the cervix usually triggers a human immune response, and in most cases HPV-infected cells are eliminated. For example, keratinocytes respond to viral infection via pattern recognition receptors (PRRs) such as toll-like receptors (TLRs) and activate innate immunity through the expression of inflammatory cytokines. On the other hand, HPV-infected cells have mechanisms to evade the human immune response (Fig. 4). For example, HPV has been reported to suppress the expression of TLR9 in infected cells [41]. Furthermore, HR-HPV-derived E6 and E7 suppress nuclear factor-kappa B (NFκB) activity, resulting in decreased expression of inflammatory cytokines [42]. In addition, E6 and E7 also inhibit type I interferon (IFN) pathways by suppressing the activation of signal transducer and activator of transcription 1 (STAT1) [43]. Regarding the IFN pathway, E6 inhibits the activation of interferon regulatory factor (IRF) 3 and E7 inhibits IFN-β production by inhibiting IRF1 [44, 45]. HPV-derived E5 and E7 suppress the ability of host cells to present HPV antigens by downregulating the expression of Human leukocyte antigen (HLA) class I, which is necessary for antigen presentation [46–48]. HPV-infected cells are recognized by antigen-presenting cells such as tissue dendritic cells (Langerhans cells in the epithelium) and tissue macrophages, and cytotoxic T cells are activated via these antigen-presenting cells. On the other hand, HPV-infected keratinocytes inhibit Langerhans cell migration by reducing the expression of macrophage inflammatory protein (MIP)-3α [49], and HR-HPV-derived E6 inhibits the differentiation of monocytes into dendritic cells [50]. In CIN and cervical cancer, the distribution of Langerhans cells has been reported to be inversely correlated with E6 and E7 expression [51, 52]. Acting as an intermediate between innate and acquired immunity, natural killer T (NKT) cells are not restricted to MHC and recognize antigens presented on CD1d and produce IFN-γ to activate antiviral immunity [53]. HPV-derived E5 functions to suppress the CD1d-presenting ability of HPV-infected cells and evade recognition by NKT cells [53].

We have described how the immune response is evaded by HPV-derived products, however, host characteristics are also associated with the immune response to HPV. In particular, the relationship between persistent HPV infection and HLA class I/II has been well studied, and the HLA class I/II polymorphisms have been reported to be associated with CIN progression [54–56]. Furthermore, loss of heterozygosity of HLA and HLA-A/B mutations are known to occur frequently in cervical cancer [5, 24]. In addition, immune cells that "suppress" tumor immunity, including regulatory T cells (Treg) and tumor-associated macrophages (TAM), are involved in CIN progression. Tregs suppress effector T cells via the production of interleukin (IL)-10 and tumor growth factor (TGF)-β [57]. Tregs are associated with the prognosis of CIN patients, with patients with high Treg counts causing persistent HPV infection and CIN [58, 59]. In addition, the presence of TAM has been reported to correlate with the prognosis of HPV-associated tumors [60, 61]. Likewise, HPV-derived products not only suppress immune responses but are also attenuated antigen-presenting capacity of HPV-infected cells. In addition to the immune escape by HPV-derived products, host characteristics including HLA polymorphisms and suppressive immune responses in the tumor microenvironment are associated with persistent HPV infection and the progression of CIN.

## Therapeutics of HPV-associated cervical neoplasia

Treatment of cervical cancer includes surgery (± adjuvant therapy), concurrent chemoradiation or chemotherapy, depending on the clinical stage of the disease. Radiation-based therapy and chemotherapy are the primary treatment strategies for advanced or recurrent cervical cancer. Although chemotherapies are not as effective for cervical cancer, the addition of bevacizumab to chemotherapies improved overall survival from 13.3 to 16.8 months [62]. Furthermore, combination therapies with immune checkpoint inhibitors (ICIs) have recently been attracting attention as a treatment option for advanced or recurrent cervical cancer, with median overall survival extended from 16.5 to 24.4 months [63]. Thereafter, combination therapy of chemotherapies and pembrolizumab was covered by insurance in Japan. In addition, genome-based medicine based on cancer genome profiling tests (CGP) has become popular recently to provide personalized medicine. However, there are still some challenges in CGP-based personalized medicine. One of the main issues is that only a limited number of patients can be enrolled in clinical trials based on genomic information [64, 65] and further improvement is desired.

Treatment strategies based on the characteristics of carcinogenic mechanisms have been investigated in HPV-associated cervical tumors. Among these, therapeutic approaches that focus on the immune response against the HPV protein have been drawing attention (Table 1). We conducted a search for recent (completed after January 2015) or ongoing Phase 2–4 clinical trials using the search terms “HPV” and “immunotherapy” on two databases, ClinicalTrial.gov (https://clinicaltrials.gov/ct2/home) and jRCT (https://jrct.niph.go.jp/). Therapeutic vaccine therapy is one of these treatments whose efficacy is expected as a possible conservative treatment. Therapeutic vaccines currently being attempted are mainly against CIN2/3, including plasmid DNA vaccines consisting of two plasmids encoding the E6 and E7 genes [66, 67] and peptide vaccines, such as oral vaccines of HPV16 E7-expressing lactobacillus [68] and HPV16-E7 synthetic long peptide (E7LP) vaccination [69]. Another immunotherapy includes engineered T-cell therapies with TCR-engineered T cells targeting E7, which are highly effective in treatment-refractory HPV-related cancers, including cervical, vulvar, anal, and oropharyngeal cancers [70]. Another therapy focusing on anti-tumor immunity is tumor-infiltrating lymphocyte therapy (TIL therapy), a type of immunotherapy in which TILs are harvested from a patient's tumor tissue, cultured outside the body, and returned to the patient. It is expected to be effective in cervical cancer [71]. Besides TIL therapies, antibody-based therapeutics are also expected to activate anti-tumor immunity. For instance, tisotumab vedotin, an investigational antibody–drug conjugate (ADC) directed against tissue factor (TF), a protein highly prevalent in multiple solid tumors, has demonstrated antitumor activity with a manageable and tolerable safety profile in women with previously treated recurrent or metastatic cervical cancer in a phase II clinical trial (NCT03438396) [72]. Additionally, as cervical cancer is caused by viral carcinogenesis, there is hope for the development of therapeutics that can trigger immune responses against both HPV and tumors in the coming years.

**Table 1:** Immune-stimulating therapies for HPV-associated cervical lesions in clinical trials

Immune-stimulating therapies *	References
Therapeutic vaccine therapy	
Plasmid DNA vaccines	[66, 67]
Peptide vaccine	[68, 69]
Engineered T-cell therapies	[70]
TIL therapy	[71]
Antibody–drug conjugates	[72]

*To note, immune checkpoint inhibitors alone are not included

The table summarizes representative immune-stimulating therapies for HPV-associated cervical cancer and precancer. These therapies were identified through searches of “ClinicalTrial.gov” and jRCT using keywords such as “Cervical cancer”, “HPV”, and “Immunotherapy” and filtered with “Phase II–IV studies” and “Interventional study”. Key terms for jRCT included “uterine cervix”, “cervical cancer”, or “HPV”

## Abbreviations

CIN: Cervical intraepithelial neoplasia
CGP: Cancer genome profiling
HLA: Human leukocyte antigen
HR-HPV: High risk human papillomavirus
HSIL: High-grade squamous intraepithelial lesion
IFN: Interferon
IRF: Interferon regulatory factor
MHLW: The Ministry of Health, Labor and Welfare
NKT: Natural killer T
SCJ: Squamocolumnar junction
TAM: Tumor-associated macrophages
TIL: Tumor-infiltrating lymphocyte
TLR: Toll-like receptors
Treg: Regulatory T cells
TZ: Transformation zone
